# Income inequality and foregone medical care in Europe during The Great Recession: multilevel analyses of EU-SILC surveys 2008–2013

**DOI:** 10.1186/s12939-016-0389-6

**Published:** 2016-07-07

**Authors:** Jon Ivar Elstad

**Affiliations:** NOVA, Centre for Welfare and Labour Research, Oslo and Akershus University College of Applied Sciences, P.O.B. 4, St. Olavs Plass, 0130 Oslo, Norway

**Keywords:** Access to healthcare, Economic crisis, Income inequality, Equity in health

## Abstract

**Background:**

The association between income inequality and societal performance has been intensely debated in recent decades. This paper reports how unmet need for medical care has changed in Europe during The Great Recession, and investigates whether countries with smaller income differences have been more successful than inegalitarian countries in protecting access to medical care during an economic crisis.

**Methods:**

Six waves of EU-SILC surveys (2008—2013) from 30 European countries were analyzed. Foregone medical care, defined as self-reported unmet need for medical care due to costs, waiting lists, or travel difficulties, was examined among respondents aged 30–59 years (*N* = 1.24 million). Countries’ macro-economic situation was measured by Real Gross Domestic Product (GDP) per capita. The S80/S20 ratio indicated the country’s level of income inequality. Equity issues were highlighted by separate analyses of disadvantaged respondents with limited economic resources and relatively poor health. Cross-tabulations and multilevel linear probability regression models were utilized.

**Results:**

Foregone medical care increased 2008—2013 in the majority of the 30 countries, especially among the disadvantaged parts of the population. For the disadvantaged, unmet need for medical care tended to be higher in countries with larger income inequalities, regardless of the average economic standard in terms of GDP per capita. Both for disadvantaged and for other parts of the samples, a decline in GDP had more severe effects on access in inegalitarian countries than in countries with less income inequality.

**Conclusions:**

During The Great Recession, unmet need for medical care increased in Europe, and social inequalities in foregone medical care widened. Overall, countries with a more egalitarian income distribution have been more able to protect their populations, and especially disadvantaged groups, against deteriorated access to medical care when the country is confronted with an economic crisis.

## Background

The “afflictions of inequality” thesis argued by Richard G. Wilkinson, Kate Pickett, and others [1–4] focuses mostly on population health, but the thesis has also been applied to a number of social ills such as low trust, crime and homicides, teenage pregnancies, and lower literary scores [2, 3]. The claim is that a society’s performance on a wide array of social outcomes will be better, the more egalitarian income distribution. The proposition that larger income inequalities tend to be associated with more social dysfunctions, at least when high-income countries are examined, has received much attention and considerable support. A recent report from Organization of Economic Cooperation and Development (OECD) has for instance linked higher income inequality to low social cohesion, lack of social mobility, and poor economic growth [5]. However, disagreements are voiced, from plain rejection (e.g. [6]) to criticism which acknowledges the empirical associations, but challenges the psychosocial interpretations given by Wilkinson and Pickett [7–10]. In addition, the thesis has been explored by numerous empirical studies (e.g. [11–16]).

The purpose of the present paper is to explore further the relationship between income inequality and societal quality, by asking whether levels of unmet medical care in Europe are associated with countries’ income differences. The context is the economic crisis which took hold in Europe in 2008. Rising unemployment and wage cuts have worsened the financial situation for many households [17, 18]. Some governments have reduced health expenditures, shut down hospitals and health centres, and increased user fees for treatment and health insurance [19–25]. The result could be increasing difficulties in obtaining medical assistance, but do such consequences depend on the country’s level of income inequality?

The present paper addresses this question by analyzing three issues. First, an account is given of self-perceived difficulties in access to medical care in Europe during The Great Recession. Previous reports on this topic indicate that an overall deterioration has occurred [26, 27], but changes do not seem dramatic, and country differences are large [22, 25, 28–30]. The present analysis covers developments in 30 European countries up to 2013. Although practically all European countries have health insurance with wide coverage [31, 32], it is well documented that low-income categories report unmet need for medical care more often than other parts of the population [27, 28, 30]. The present study expands on previous analyses by drawing attention to the experiences of those who are especially vulnerable since they combine two unfavourable characteristics: limited economic resources and considerable need for medical care. Good access to health services for such disadvantaged parts of the population will be an important performance indicator for a healthcare system.

Second, associations between self-perceived unmet need for medical care and income inequalities are examined. The “afflictions-of-inequality” thesis contends that in economically advanced countries, social well-being and societal performance do not necessarily vary with the average economic level, but primarily with the magnitude of income inequalities [3]. Accordingly, we ask whether higher income inequality in the analyzed 30 countries goes together with higher levels of unmet medical care, after adjusting for the country’s Real Gross Domestic Product per capita.

Third, the income inequality issue is pursued further by asking whether countries with a more egalitarian income distribution have been better able to protect access to medical care when the country is drawn into an economic crisis. In today’s global capitalism, narrow income differences in themselves will hardly protect against recessions and economic downturns. Most national economies will be vulnerable to contractions in international markets – not the least the small and “open” economies of many European countries. Accordingly, the third research question is not whether income equality buffers against a fall in the country’s national product, but whether egalitarian countries, when experiencing an economic downturn, succeed better in safeguarding access to medical care than more inegalitarian countries. Negative macro-economic developments will probably tend to be followed by deteriorated access to medical care, but the issue here is whether the negative impact of an economic downturn of a given size is smaller in more egalitarian countries.

## Methods

### Data, variables, samples

The study utilized survey data from the European Union Statistics on Income and Living Conditions program (EU-SILC). EU-SILC provides rich information from large population samples, obtained through harmonized surveys conducted yearly [33, 34]. Six survey rounds (2008–2013) with data from 30 countries were analyzed (all European Union (EU) member states except Croatia, plus the three non-member countries Iceland, Norway, and Switzerland). Individuals aged 30 to 59 years were selected for the analyses. The very few respondents with missing values on relevant variables were excluded from the analyzed samples which number more than one million respondents (*N* = 1,242,361).

In the EU-SILC surveys, respondents were asked whether they “really needed” medical examination or treatment, but did not get it on at least one occasion during the last 12 months. If “yes”, respondents were asked to state the reason by choosing from a list of eight alternatives [33]. The dichotomous outcome analyzed in this study, *foregone medical care*, indicates self-reported unmet need for medical care either because of costs, waiting lists, or travel difficulties. This variable, also used in previous research [e.g. 28, termed “enforced unmet need”], is likely to reflect difficulties in access to medical care due to circumstances associated with an economic crisis, such as austerity, insufficient supply of health care, higher co-payments, and lack of household economic resources. Among the three alternative reasons for foregone medical care, “too expensive” was the most common [35].

Other individual-level variables were gender, age, educational level (three categories low, medium, and high), and total disposable household income [33]. Real Gross Domestic Product (GDP) per capita [36] was used for characterizing the country’ overall economic level, and change in this variable indicates macro-economic growth or decline. Income inequality was indicated by Eurostat’s calculations of the S80/S20 ratio, i.e., the ratio of total income received by the 20 % of the population with the highest income (top quintile), to the total income of the fifth with lowest income (lowest income quintile) [37, 38].

As equity in health care is a central issue, foregone medical care among less privileged population categories was especially examined. Each country sample was therefore divided into two parts. The *Disadvantaged* were defined by two criteria: they were in the lowest income tertile (the lower third of the income distribution in the country sample, age 30–59, in the given survey), and they reported health difficulties in terms of either a long-standing (chronic) disease or self-rated overall health status as fair or bad. Reports and statistics (see, for instance, [27]) indicate that in almost all European countries, foregone medical care is seldom reported by middle-income and high-income individuals [31]. Moreover, although everybody will need medical assistance from time to time, delays in obtaining medical care may be quite unproblematic for those in good health. A crucial test, so to speak, for a healthcare system is therefore whether people with limited economic resources whose well-being depends on access to medical services, have few difficulties in obtaining medical care. The category *Disadvantaged* is intended to represent this vulnerable part of the population, in so far as possible with the available information in the EU-SILC surveys. However, also the remaining parts of the samples, termed *Others*, were analyzed in order to address more broadly how foregone medical care has developed in European populations during The Great Recession.

### Analyses

First, data were described, and percentages reporting foregone medical care in each country, both among Disadvantaged and Others, are shown. Since sample distributions could vary randomly from year to year, the surveys were pooled in pairs. Changes during The Great Recession were indicated by the 2008/2009 and 2012/2013 years, i.e. roughly the pre-crisis and “peak-of-crisis” situation. The economic downturn started earlier in some countries (e.g., Iceland) than in others (e.g., Greece) [36]. The EU-SILC surveys indicate that foregone medical care was at its lowest level in 2009 [27].

Second, three-level regression models were analyzed, which correspond to the hierarchical data structure: cross-sectional survey samples (individual level), conducted at different time-points (level of country-years), nested within countries (country level). The country level variables were coded as centred means for the period under study, while the country-years variables measured deviations from these country-specific means at different time points. This allows for simultaneous estimations of the effects of both time-invariant and time-varying country characteristics (see [39, 40] and [41–44]). Linear probability regression was used. Logistic regression is often preferred for dichotomous outcomes, as it avoids some difficulties with linear probability models, but the two techniques give mostly very similar findings [45]. Linear probability models have, on the other hand, some advantages. Results are more easily interpreted than logits and odds ratios, as the linear probability coefficients represent the change in *proportions* (or percentages) reporting foregone medical care given a one-unit increase in the explanatory variable. Moreover, statistical packages have often difficulties in fitting logistic models in large samples, especially when three levels (as in this study) have to be handled.

At the country level, two variables with constant values during the study period were used: Average Real GDP per capita 2007–2012 [36], measured in fixed Euros and recalculated into natural logarithms, and average income inequality, i.e. the mean S80/S20 ratio 2007–2012 [38]. Both variables were centred around the mean value for the 30 countries.

At the next country-years level the variable GDP deviation indicated how much the country’s GDP deviated from the country average 2007—2012 at three time points: 2007/2008, 2009/2010, and 2011/2012. The unit of this variable was ten percent *decline* in GDP per capita, implying, for example, that a value of +0.8 for 2011/2012 means that Real GDP per capita was eight percent *lower* than the 2007—2012 average. Thus, the coefficient for this variable indicates how much the proportion experiencing foregone medical care could be expected to change if GDP per capita decreased by 10 %. The data file was arranged so that GDP deviation in 2007/2008 corresponded to the individual-level information in 2008/2009, and so on (i.e. GDP deviation 2011/2012 corresponded to the 2012/2013 surveys). The rationale for this was an assumption about a likely time lag of about 1 year between a macro-economic change and possible effects on access to medical care.

Similarly, the variable Income inequality deviation measured how much the S80/S20 ratio in 2007/2008, 2009/2010, and 2011/2012, respectively, deviated from the average S80/S20 ratio for the 2007—2012 period.

The outcome variable foregone medical care was measured at the individual level (for 2008/2009, 2010/2011, and 2012/2013), and at this level there are also individual controls: gender (women = 1), age (in years, centred around the midpoint 45 years = 0), age squared, dummy variables for low and high education (reference = medium education), and two dummy variables which indicated whether the respondent was unemployed at the time of interviewing, and whether he/she lived in a rural area.

Only results from models relevant for the research questions outlined in the Background section are reported. The second issue was examined by analyzing whether a more egalitarian income distribution during 2007–2012 was associated with lower levels of foregone medical care (less unmet need for medical assistance) in the 30 analyzed countries, independent of how “rich” the country was. Thereafter, the third issue – whether a more egalitarian income distribution moderated negative effects on foregone medical care of an economic downturn – was investigated. Here, the crucial question was whether the *interaction* between change in GDP and income inequality had any effect on foregone medical care.

Intercept variances and random slope variances at the country level for gender, age, and low education were estimated [44]. To include other random slope effects was not feasible since they resulted in non-converging models. Estimations were made by means of MLwiN, version 2.32, run from within Stata [46].

## Results

### Descriptive analyses

Table 1 presents the samples and reports some country-level indicators. On average, 14.7 % of the survey samples aged 30–59 were classified as Disadvantaged, varying from 9.3 % in Romania to 22.2 % in Latvia. This percentage varied little within each country during the study period. The level of income inequality was indicated by the average S80/S20 ratio during 2007–2012, which varied from lowest (3.30) in Slovenia to 7.27 in Latvia. Changes in the S80/S20 ratio during 2007–2012 were quite small (not shown in table). Average Real GDP per capita varied about ten-fold, from lowest in Bulgaria and Romania (less than 6,500 Euros) to highest in Switzerland, Norway, and Luxembourg. The impact of The Great Recession is indicated in the right-hand column which shows that Real GDP per capita declined from 2007/2008 to 2011/2012 in 21 of the 30 countries. In the “declining” countries, GDP was usually reduced from two to ten per cent (16 countries); Greece was exceptional with a GDP decline of 22 %. Only Poland had a substantial GDP increase from 2007/2008 to 2011/2012.

**Table 1:** Descriptive data. Number of respondents, Disadvantaged and Others, aged 30–59, EU-SILC surveys 2008—2013. Income inequality and GDP per capita, 30 countries

30 countries, ranked by level of income inequality	Number of respondents		Average S80/S20 ratio 2007–2012	Real GDP per capita (Euros per capita)	
	Disadvantaged	Others		Average 2007–2012	Change 7/8–11/12
Slovenia	4770	23,629	3.30	18,017	92.9
Norway	2661	14,968	3.52	67,250	96.4
Sweden	2680	16,795	3.57	39,467	99.5
Czech Republic	6363	37,090	3.65	15,050	98.7
Finland	5345	25,687	3.70	35,650	94.6
Iceland	1086	8381	3.72	32,767	93.7
Slovakia	5758	33,541	3.78	12,417	105.3
Hungary	12,441	53,677	3.90	10,017	96.1
Netherlands	4870	29,356	3.93	38,400	97.6
Belgium	4974	30,700	3.97	33,667	99.4
Malta	3081	22,670	4.12	15,933	103.8
Austria	5301	30,232	4.15	35,667	100.7
Cyprus	3017	23,725	4.22	23,217	91.0
Luxembourg	4407	29,119	4.25	78,400	93.9
Denmark	2404	14,904	4.32	44,317	95.1
France	9834	52,865	4.35	31,067	99.2
Switzerland	5039	33,940	4.43	56,117	100.1
Ireland	3392	23,972	4.65	37,833	93.4
Germany	11,148	56,631	4.73	31,833	103.0
Poland	15,354	72,171	5.30	9317	113.7
United Kingdom	6467	36,458	5.42	29,467	96.7
Estonia	5179	20,779	5.53	12,033	94.6
Italy	12,805	107,919	5.77	27,183	93.0
Portugal	6858	26,625	6.07	16,817	95.3
Spain	10,072	79,656	6.27	23,433	92.4
Greece	6536	33,296	6.42	20,467	78.8
Bulgaria	4120	30,972	6.62	5100	104.0
Lithuania	5391	20,773	6.68	9617	101.0
Romania	4305	42,139	7.12	6367	101.1
Latvia	7548	26,485	7.27	9350	94.0

Change 7/8–11/12 = Real GDP per capita in 2011/2012, as a percentage of GDP in 2007/2008

The correlation coefficients (Pearson’s r) between the country indicators (not shown in table) provide interesting information. “Richer” countries tended to have less income inequality: The correlation between average GDP per capita and average S80/S20 ratio 2007–2012 was −0.49 (*p*-value <0.01). Income inequality was on the other hand practically unrelated to GDP change from 2007/2008 to 2011/2012 (*r* = 0.09, *p*-value 0.65). The correlation between average GDP and GDP change was insignificantly negative (−0.19, *p*-value 0.31). Thus, neither egalitarian countries nor “rich” countries seemed to be more successful in avoiding GDP decline during the crisis years than other European countries.

**Fig. 1:**
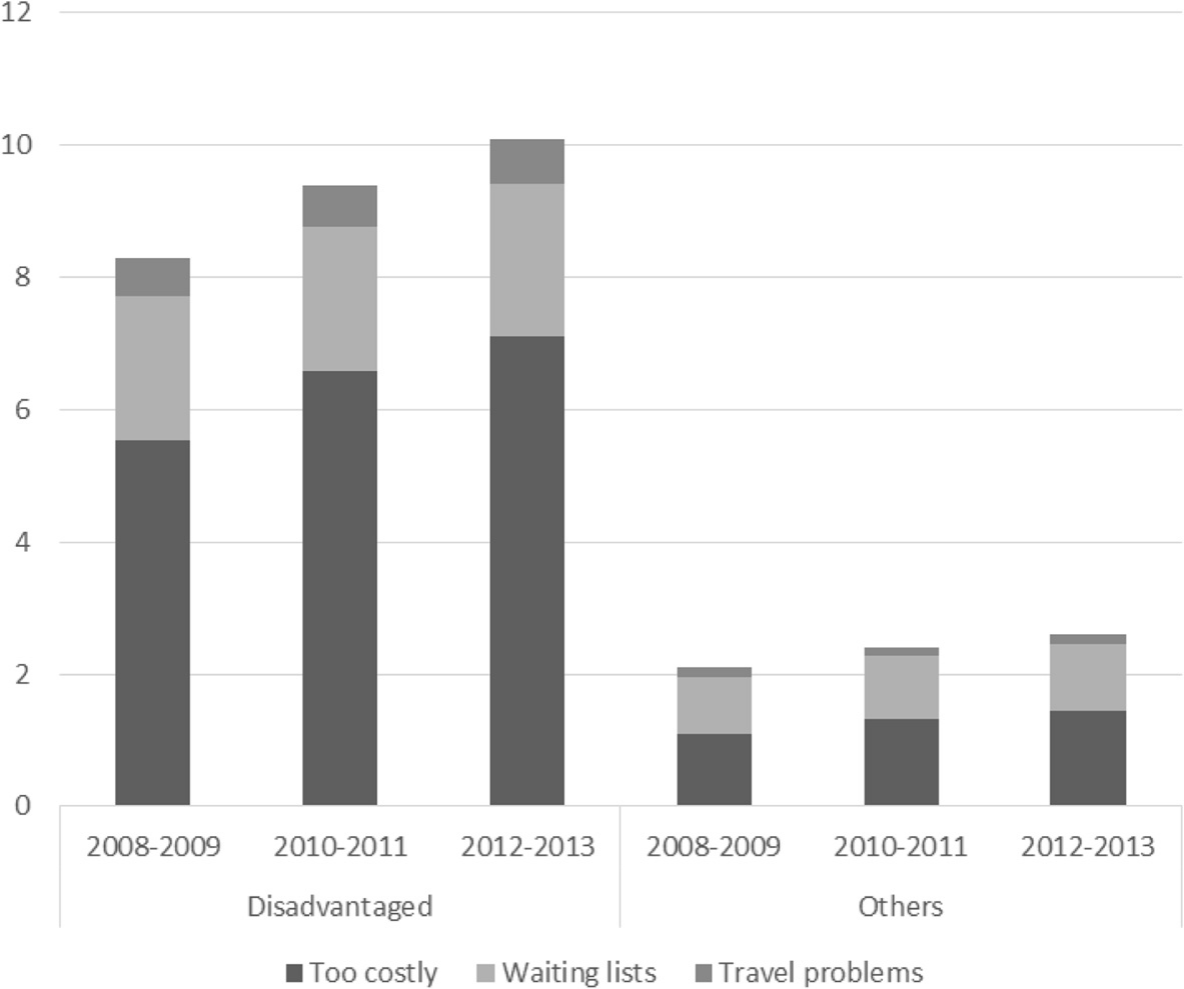
Foregone medical care (%), according to reason for unmet medical care, three periods, Disadvantaged and Others, unweighted country average

Figure 1 and Table 2 display the reporting of foregone medical care in the Disadvantaged and Others subsamples. Among the Disadvantaged, the percentage reporting foregone medical care increased from 8.3 to 10.1 % during The Great Recession (unweighted country average), while it rose from 2.1 to 2.6 % among Others (Fig. 1). Reporting foregone medical care because it was perceived as “too costly” was especially prevalent among Disadvantaged. Table 2 gives country-specific information. Two well-known patterns [27, 28, 30, 31] can be noted – the huge between-country differences, and the ubiquitous social inequalities. In 2012/2013, for example, foregone medical care in the Disadvantaged subsamples varied from 0.1 % in Slovenia to 35.4 % in Romania. Moreover, in every country and in both periods, percentages reporting foregone medical care were higher (sometimes very much higher) among Disadvantaged than Others – only one exception: Slovenia in 2012/2013.

**Table 2:** Foregone medical care (%) in 2008/2009 and 2012/2013, Disadvantaged and Others subsamples, 30 countries

	Disadvantaged				Others			
Country	2008/2009	2012/2013	Change	Sig.	2008/2009	2012/2013	Change	Sig.
Belgium	2,3	10,7	8,4	**	0,3	1,2	0,9	**
Iceland	7,0	14,3	7,3	**	1,4	2,8	1,4	**
Italy	15,8	22,8	7,0	**	3,6	4,6	1,0	**
Greece	13,4	20,0	6,6	**	2,8	5,5	2,7	**
Latvia	24,3	30,8	6,5	**	6,1	9,6	3,5	**
Poland	15,3	19,5	4,2	**	4,8	6,1	1,3	**
Cyprus	12,5	15,7	3,2	*	2,5	3,5	1,0	**
Finland	4,2	7,3	3,1	**	1,5	2,8	1,4	**
Ireland	4,7	7,7	3,0	**	1,7	3,6	1,9	**
Portugal	6,6	8,9	2,4	**	1,8	2,6	0,9	**
Estonia	9,8	12,2	2,3	*	4,9	7,2	2,3	**
Slovakia	3,1	5,4	2,3	**	1,0	1,1	0,2	
Denmark	2,2	3,9	1,7	*	0,6	0,8	0,2	
Czech Republic	1,6	3,2	1,6	**	0,4	0,4	0,0	
Hungary	8,9	10,4	1,5	*	1,8	1,6	−0,2	
France	6,0	7,3	1,4	*	1,3	1,7	0,4	**
Spain	1,3	2,4	1,1	**	0,3	0,6	0,2	**
Norway	3,2	4,1	0,9		0,8	1,1	0,3	
Switzerland	3,3	3,9	0,6		0,8	0,8	0,0	
Malta	3,0	3,4	0,4		0,8	1,0	0,2	
Netherlands	1,4	1,7	0,4		0,2	0,3	0,1	
United Kingdom	2,9	2,9	0,0		0,9	1,5	0,5	**
Luxembourg	3,8	3,5	−0,2		0,5	0,6	0,1	
Austria	2,2	1,7	−0,5		0,4	0,2	−0,2	
Slovenia	0,8	0,1	−0,7		0,1	0,1	0,0	
Romania	36,3	35,4	−0,8		4,8	5,7	0,8	**
Germany	6,8	5,6	−1,2		1,1	0,9	−0,2	
Sweden	7,4	5,3	−2,1		1,7	1,2	−0,5	
Lithuania	7,5	5,2	−2,3		3,4	1,9	−1,5	
Bulgaria	32,4	27,1	−5,4		10,0	6,1	−3,9	
Unweighted country average	8.3	10.1	1.8		2.1	2.6	0.5	

Sig. = significance of *increase* in foregone medical care (one-sided test): ** = *p*-val < 0.01. * = *p*-val < 0.05. Due to rounding, figures in Change column deviate sometimes from 2012/2013 minus 2008/2009 value

For this paper, the *changes* during the crisis years are the central interest, however. Figure 1 and Table 2 indicate both deteriorated access and increasing social inequalities during The Great Recession. Among Disadvantaged, foregone medical care increased in 21 countries (significantly, i.e., *p*-values < 0.05, in 17 countries), was stable in United Kingdom, and decreased in eight countries. Among Others, foregone medical care also increased in 21 countries (although often marginally), while three countries saw no changes, and foregone medical care decreased, usually just a little, in six countries. Table 2 reveals larger increases in foregone medical care (in percentage points) among Disadvantaged than among Others in the majority of the countries. The differences between Disadvantaged and Others increased by more than 0.5 percentage points in 18 of the 30 countries, decreased by at least 0.5 points in seven countries, and remained relatively stable in five countries.

### Multilevel analyses

Table 3 reports the results from the multilevel analyses of the Disadvantaged subsamples. As the individual-level coefficients are of less interest for this paper, comments will focus on the country and country-years variables.

**Table 3:** Foregone medical care, three-level linear probability models, Disadvantaged respondents (*N* = 183,206, 90 country-years, 30 countries)

	Model 1			Model 2			Model 3		
Fixed effects	Coeff.	SE	Sig	Coeff.	SE	Sig	Coeff.	SE	Sig
Intercept	0.087	0.0108	***	0.087	0.0108	***	0.087	0.0108	***
Women	0.016	0.0027	***	0.016	0.0027	***	0.016	0.0027	***
Age centred	−0.000	0.0002	*	−0.000	0.0002	*	−0.000	0.0002	*
Age centred squared	−0.000	0.0000	***	−0.000	0.0000	***	−0.000	0.0000	***
Low education	0.020	0.0064	**	0.020	0.0064	**	0.020	0.0064	**
High education	−0.009	0.0021	***	−0.009	0.0021	***	−0.009	0.0021	***
Unemployed	0.054	0.0018	***	0.054	0.0018	***	0.054	0.0018	***
Rural area	−0.016	0.0015	***	−0.016	0.0015	***	−0.016	0.0015	***
GDP level (log)	−0.033	0.0153	*	−0.033	0.0153	*	−0.033	0.0153	*
Income inequality level	0.024	0.0089	**	0.024	0.0089	**	0.024	0.0089	**
GDP deviation				0.015	0.0059	**	0.011	0.0061	ns
Income ineq. deviation				0.007	0.0079	ns	0.002	0.0079	ns
GDP deviation * income inequality level							0.010	0.0047	*
Random intercept	Variance	95 % conf. interval		Variance	95 % conf. interval		Variance	95 % conf. interval	
Individual level	0.0805	0.0800	0.0810	0.0805	0.0800	0.0810	0.0805	0.0800	0.0810
Country-years level	0.0004	0.0002	0.0005	0.0003	0.0002	0.0004	0.0003	0.0002	0.0004
Country level	0.0032	0.0015	0.0049	0.0032	0.0015	0.0049	0.0032	0.0015	0.0050
Random slopes									
Women	0.0002	0.0000	0.0003	0.0002	0.0000	0.0003	0.0002	0.0000	0.0003
Age centred	0.0000	0.0000	0.0000	0.0000	0.0000	0.0000	0.0000	0.0000	0.0000
Low education	0.0011	0.0005	0.0017	0.0011	0.0005	0.0017	0.0011	0.0005	0.0017
−2LL	58,716.78			58,706.61			58,702.24		

SE = standard error. Sig.: *** = *p*-val < 0.001. ** = *p*-val < 0.01. * = *p*-val < 0.05. ns = not significant. Random slopes refer to country level

In Model 1, the individual-level predictors (both as fixed and random effects), the country’s GDP level (natural logarithms of Real GDP per capita, average 2007–2012), and country level of income inequality (S80/S20 ratio, average 2007–2012), were included. This model indicated a significant effect (using the *p* < 0.05 criterion) of the country’s average economic level: For each log unit increase in Real GDP per capita, the proportion Disadvantaged reporting foregone medical care could be expected to fall by 0.033, i.e. 3.3 percentage points. The income inequality coefficient (0.024, *p*-value < 0.01) indicated moreover that, adjusted for respondents’ characteristics and average GDP, income inequality was markedly associated with the level of foregone medical care during these years. For each step “up” on the scale of income inequality, the percentage among Disadvantaged who experienced foregone medical care could be expected to become 2.4 points higher.

Model 2 added the country-years variables, i.e., GDP and income inequality deviations from the country means at the three time points. This model indicated whether *changes* in GDP and/or income inequality had any impact on the level of foregone medical care, net of the impact of the 2007–2012 *mean* of these variables. The effect of Income inequality deviation was insignificant, but the GDP deviation coefficient (0.015, *p*-value < 0.01) indicated that overall, a 10 % decline in GDP corresponded to an increase in foregone medical care among the Disadvantaged of 1.5 percentage points.

Lastly, Model 3 added the interaction variable between GDP deviation and the 2007–2012 income inequality. The coefficient (0.010, *p*-value < 0.05) was in line with the expectation that higher income inequality aggravates the detrimental effects of an economic downturn on foregone medical care. The effect size can be illustrated as follows: If GDP declined by 10 %, foregone medical care could be expected to rise among the Disadvantaged by 1.1 percentage points in countries with medium income inequality (i.e. an S80/S20 ratio of about 4.4). Foregone medical care would however rise by around 3.1 percentage points in inegalitarian countries with S80/S20 ratios around 6.4, but the increase could be expected to be only around 0.1 percentage points in egalitarian countries with S80/S20 ratios around 3.4.

In Table 4, the results from identical multilevel models among Others are reported. The large number of Others (1,059,155 respondents) could not be handled by the available MLwiN version, and a random sample of 400,000 Others was selected, as trials showed that the statistical software could handle this sample size. In Table 4, the impact of country-level characteristics on foregone medical care appears as different among Others than for the Disadvantaged subsamples. The coefficient for GDP level was, in all three models, +0.007, implying a statistically significant, but weak and *surprising* tendency that foregone medical care among Others occurred more frequently the higher the country’s GDP per capita. The effect of a decline in GDP per capita (the variable GDP deviation) was, on the other hand, as expected and indicated that also for Others, an economic downturn would tend to be followed by rising levels of foregone medical care – but the effect was only around half as large as among the Disadvantaged. The countries’ income inequality level 2007–2012 appeared to have no overall effect, but that income inequality could be unfavourable also for Others was suggested by the significant (but small) effect of the interaction between GDP deviation and income inequality level (Model 3). When going through an economic downturn, it seems therefore that also among Others, the consequences for access to medical care would be more detrimental the larger income differences in the country.

**Table 4:** Foregone medical care, three-level linear probability models, Others (*N* = 400,000, 90 country-years, 30 countries)

	Model 1			Model 2			Model 3		
Fixed effects	Coeff.	SE	Sig	Coeff.	SE	Sig	Coeff.	SE	Sig
Intercept	0.021	0.0038	***	0.021	0.0038	***	0.021	0.0038	***
Women	0.007	0.0015	***	0.007	0.0015	***	0.007	0.0015	***
Age centred	0.000	0.0001	**	0.000	0.0001	**	0.000	0.0001	**
Age centred squared	0.000	0.0000	ns	0.000	0.0000	ns	0.000	0.0000	ns
Low education	0.013	0.0044	**	0.013	0.0044	**	0.013	0.0044	**
High education	−0.006	0.0006	***	−0.006	0.0006	***	−0.006	0.0006	***
Unemployed	0.026	0.0010	***	0.026	0.0010	***	0.026	0.0010	***
Rural area	−0.007	0.0006	***	−0.007	0.0006	***	−0.007	0.0006	***
GDP level (log)	0.007	0.0019	***	0.007	0.0019	***	0.007	0.0019	***
Income inequality level	−0.000	0.0011	ns	−0.000	0.0011	ns	−0.000	0.0011	ns
GDP deviation				0.008	0.0023	***	0.006	0.0024	*
Income ineq. deviation				0.003	0.0031	ns	0.001	0.0031	ns
GDP deviation * income inequality level							0.004	0.0018	*
Random intercept	Variance	95 % conf. interval		Variance	95 % conf. interval		Variance	95 % conf. interval	
Individual level	0.0234	0.0233	0.0235	0.0234	0.0233	0.0235	0.0234	0.0233	0.0235
Country-years level	0.0001	0.0000	0.0001	0.0001	0.0000	0.0001	0.0001	0.0000	0.0001
Country level	0.0004	0.0002	0.0006	0.0004	0.0002	0.0006	0.0004	0.0002	0.0006
Random slopes									
Women	0.0001	0.0000	0.0001	0.0001	0.0000	0.0001	0.0001	0.0000	0.0001
Age centred	0.0000	0.0000	0.0000	0.0000	0.0000	0.0000	0.0000	0.0000	0.0000
Low education	0.0006	0.0003	0.0009	0.0006	0.0003	0.0009	0.0006	0.0003	0.0009
−2LL	−365,942.31			−365,958.19			−365,963.56		

The sample of 400,000 was selected randomly from the original 1,059,155 respondents in the Others samples. SE = standard error. Sig.: *** = *p*-val < 0.001. ** = *p*-val < 0.01. * = *p*-val < 0.05. ns = not significant. Random slopes refer to country level

## Discussion

### Main results and possible interpretations

With respect to the research issues addressed by this paper, the findings can be summarized as follows. First, although there are many variations between the 30 analyzed European countries, the overall tendency during the crisis years was deteriorated access to medical examination and treatment, i.e. rising levels of self-reported foregone medical care among the 30–59 years olds examined in this study. This negative development was markedly stronger among disadvantaged respondents (those with both relatively low income and health problems) than among other parts of the samples. Thus, increasing levels of foregone medical care, as well as increasing social *inequalities* in foregone medical care, are noticeable and worrying facets of The Great Recession in Europe.

Second, independent of the country’s overall economic level (measured by GDP per capita), larger income inequalities were clearly associated with more reporting of foregone medical care during the 2008—2013 period among the disadvantaged subsamples (which constituted, on average, about 15 % of the country samples). This unfortunate association between income inequality and access occurred only among the disadvantaged, however, and the association was not found for the other parts of the analyzed samples.

Third, foregone medical care tended to deteriorate with economic downturns, but such adverse developments were stronger the larger the country’s income inequality. Although small income inequalities did not protect against an economic crisis as such (i.e. against GDP decline), a more egalitarian income distribution was associated with less negative effects on access to medical care when the country endured an economic crisis. In this sense, smaller income inequalities seemed beneficial for protecting access during crisis times – in particular for disadvantaged people, but also for other parts of the population.

In sum, the findings of this study correspond, at least to a considerable extent, to the “afflictions of inequality” thesis: Independent of the average affluence of a country (as measured by GDP per capita), social dysfunctions and unfortunate societal characteristics tend to be worse the larger income inequality in the country. Access to medical care (as experienced by the population) may be added to the list of such unfortunate societal aspects – but what processes and mechanisms could generate this association?

Status anxiety and psychosocial injuries, often invoked for explaining associations between income inequality and inhabitants’ health and well-being [4], seem less relevant for the topic examined by the present study. More relevant is probably the obvious difference between egalitarian and inegalitarian countries. In the latter, a larger part of the population will typically have incomes far below the average. Therefore, the proportion of the population who are sensitive to changes which worsen access may be larger in inegalitarian countries. Barriers to obtaining medical care will typically increase during an economic crisis, because supply is reduced (e.g. closure of health facilities), utilization becomes more costly (e.g. increased co-payments), or households’ economic situation worsens [19, 21, 24]. The larger the proportion of the population who are likely to be influenced by such changes, the larger negative effect on foregone medical care is likely to occur. One explanation for the findings in this study, for instance that foregone medical care increased more among Disadvantaged than among Others, could therefore be that the proportion in the population “at risk” for social misfortunes are typically larger in inegalitarian countries.

This “mechanical” explanation can be challenged, however, by an interpretation which highlights political factors. If policies aiming at limiting income inequalities have wide support, governing politicians may also be more inclined to prevent both reduced access to medical services and larger social inequalities in access. This could imply that proposals for increasing user fees will be rejected, exemptions from co-payments will be more generous, and health budgets will suffer less cuts – and, in general, that the healthcare system is more oriented towards safeguarding access.

This explanation is in line with interpretations of the associations between income inequality and social ills which emphasize that political power is consequential both for income inequality, the generosity of welfare services [7–9, 47, 48], and for how social policies influence access to medical care [30]. However, the findings in this study cannot be explained simply by types of welfare regimes [49, 50]. Analyses of the Disadvantaged samples (Table 3, Model 3) with fewer countries – first, only the 25 non-Nordic countries, and second by excluding Spain, Italy, and Greece and analyzing the remaining 27 countries – gave coefficients for the income inequality variables very similar to those reported in Table 3.

The socio-political approach could be interpreted as implying that income inequality is primarily an outcome of more fundamental social and economic forces, without a causal impact “on its own”. This conclusion may be challenged, however. Smaller income inequalities in a country may enhance social solidarity and social cohesion, and these factors may in themselves facilitate policies which protect access to medical care in times of crisis.

### Limitations

A potential problem is the representativity of the surveys [51], but it seems unlikely that the results are grossly misleading because of sample bias. To what extent the questions used by EU-SILC provide appropriate measurements of accessibility to medical care is debated [52]. One problem which has been given little attention is that measuring access by self-reports of foregone medical care may potentially underestimate access difficulties. When afflicted by severe medical conditions, most people will somehow manage to get medical assistance, no matter how much they have to pay or how far they have to travel, because abstention is not a viable option. Thus, they have not abstained from medical care and may decline to report foregone care, regardless of how difficult access actually was.

The results of multilevel analyses may depend on how the models are designed [44]. The somewhat surprising result that among Others, levels of foregone care were higher the higher the GDP level in the country (Table 4), appeared only when the individual-level variables gender, age, and low education, were included as random slope effects. Often, multilevel studies take only random intercepts into account, and if the present study had dropped random slopes for the individual-level variables, GDP level and income inequality would have had significant effects on foregone medical care also in the Others subsamples, although weaker than the effects observed among Disadvantaged.

A limitation in this study may be that only two country-level characteristics – GDP per capita, and income inequality – were analyzed. More country-level indicators might give a better understanding of processes and mechanisms, but the limited number of higher-level units (30 countries) limits the number of country-level indicators which could be analyzed.

This study has in particular examined a subsample termed Disadvantaged, motivated by the argument that their access will be of considerable health policy interest and particularly relevant for judging the performance of the healthcare system. An objection could be that the Disadvantaged are relatively few, making it dubious to use their experiences as a basis for more general assessments. This objection is relevant, but it should be noted that although the Disadvantaged constitute only 10–20 % of the samples, they stand for 41.0 % (in the entire sample analyzed here) of all those who reported foregone medical care.

## Conclusions

Access to medical care has deteriorated in the majority of European countries during The Great Recession, in particular among disadvantaged parts of the population. Independent of the overall economic level in the country (measured by GDP per capita), access was more problematic the higher income inequality in the country, in particular among disadvantaged sections of the population. When going through an economic downturn of a given severity, access deteriorated more the larger income differences in the country.

## Abbreviations

EU-SILC, European Union Statistics on Income and Living Conditions; EU, European Union; GDP, gross domestic product; S80/S20 ratio, ratio of total income received by the top income quintile, to that received by the bottom income quintile; OECD, organization of economic cooperation and development.
